# Super-Resolution Microscopy Reveals Local Accumulation of Plasma Membrane Gangliosides at *Neisseria meningitidis* Invasion Sites

**DOI:** 10.3389/fcell.2019.00194

**Published:** 2019-09-13

**Authors:** Jan Schlegel, Simon Peters, Sören Doose, Alexandra Schubert-Unkmeir, Markus Sauer

**Affiliations:** ^1^Department of Biotechnology and Biophysics, Biocenter, Julius Maximilian University Würzburg, Würzburg, Germany; ^2^Institute of Hygiene and Microbiology, Julius Maximilian University Würzburg, Würzburg, Germany

**Keywords:** *Neisseria meningitidis*, sphingolipids, gangliosides and lipid rafts, super-resolution microscopy, single-molecule tracking

## Abstract

*Neisseria meningitidis* (meningococcus) is a Gram-negative bacterium responsible for epidemic meningitis and sepsis worldwide. A critical step in the development of meningitis is the interaction of bacteria with cells forming the blood-cerebrospinal fluid barrier, which requires tight adhesion of the pathogen to highly specialized brain endothelial cells. Two endothelial receptors, CD147 and the β2-adrenergic receptor, have been found to be sequentially recruited by meningococci involving the interaction with type IV pilus. Despite the identification of cellular key players in bacterial adhesion the detailed mechanism of invasion is still poorly understood. Here, we investigated cellular dynamics and mobility of the type IV pilus receptor CD147 upon treatment with pili enriched fractions and specific antibodies directed against two extracellular Ig-like domains in living human brain microvascular endothelial cells. Modulation of CD147 mobility after ligand binding revealed by single-molecule tracking experiments demonstrates receptor activation and indicates plasma membrane rearrangements. Exploiting the binding of Shiga (STxB) and Cholera toxin B (CTxB) subunits to the two native plasma membrane sphingolipids globotriaosylceramide (Gb3) and raft-associated monosialotetrahexosylganglioside GM1, respectively, we investigated their involvement in bacterial invasion by super-resolution microscopy. Structured illumination microscopy (SIM) and *direct* stochastic optical reconstruction microscopy (*d*STORM) unraveled accumulation and coating of meningococci with GM1 upon cellular uptake. Blocking of CTxB binding sites did not impair bacterial adhesion but dramatically reduced bacterial invasion efficiency. In addition, cell cycle arrest in G1 phase induced by serum starvation led to an overall increase of GM1 molecules in the plasma membrane and consequently also in bacterial invasion efficiency. Our results will help to understand downstream signaling events after initial type IV pilus-host cell interactions and thus have general impact on the development of new therapeutics targeting key molecules involved in infection.

## Introduction

The obligate human pathogenic Gram-negative bacterium *Neisseria meningitidis* can cause epidemic meningitis and in severe cases sepsis and progressing fatal shock (Stephens et al., 2007). In healthy individuals the meningococci may reside as commensal organisms in the nasopharynx without affecting the host (Yazdankhah and Caugant, 2004). Under certain circumstances, the bacteria can enter the bloodstream and adhere to endothelial cells of blood microvessels, also known as vascular colonization (Melican and Dumenil, 2012) leading to inflammatory processes and disruption of the blood-cerebrospinal fluid barrier, a crucial step in disease progression into meningitis (reviewed in Lemichez et al., 2010). The initial process of bacterial adhesion to brain endothelial cells is mediated by type IV pili and its adhesion receptor CD147 on the host cell (Bernard et al., 2014).

Recently, super-resolution microscopy by *direct* stochastic optical reconstruction microscopy (*d*STORM) (Heilemann et al., 2008) demonstrated that *N. meningitidis* binding to endothelial cells requires CD147/β2-adrenergic receptor clustering at bacterial adhesion sites (Maïssa et al., 2017). Here, the assembly of plasma membrane receptors might serve as platform to support host-pathogen interactions. However, the molecular process of subsequent barrier-crossing is still under debate. Besides the investigated loosening of endothelial tight junctions (Coureuil et al., 2009; Schubert-Unkmeir et al., 2010) there is evidence that meningococci may use transcytotic pathways to enter perivascular tissues (Nikulin et al., 2006; Sutherland et al., 2010). Since signaling and interactions of CD147 is dependent on plasma membrane cholesterol (Wu et al., 2017) and ganglioside-enriched lipid rafts (Li et al., 2013) downstream rearrangement of the plasma membrane might facilitate bacterial invasion of cells.

Indeed, recent data suggests meningococcal type IV pili dependent binding to gangliosides (Mubaiwa et al., 2017), which has already been known for several pathogens colonizing the respiratory tract (Krivan et al., 1988). Glycosphingolipids in general are important host cell targets for a plenitude of pathogens such as fungi, bacteria, and viruses (Nakayama et al., 2018). They are composed of complex, highly variable glycan moieties linked to a lipophilic ceramide backbone with extensive molecular heterogeneity (Lingwood, 2011). Two well studied glycosphingolipids with receptor functions are the monosialotetrahexosylganglioside GM1, a prototype ganglioside, and the globotriaosylceramide Gb3, which both interact with protein receptors within lipid rafts to generate signaling platforms (Mutoh et al., 1995; Ichikawa et al., 2009; Lingwood et al., 2010; Prasanna et al., 2016).

Besides its importance in neuronal plasticity, GM1 can be targeted by Simian virus 40 (Tsai et al., 2003), *Brucella suis* (Naroeni and Porte, 2002), Cholera toxin B subunit (Cuatrecasas, 1973), *Escherichia coli* enterotoxin (Hyun and Kimmich, 1984), and *Vibrio cholerae* enterotoxin (Otnaess et al., 1983). Gb3, also known as CD77, is a marker for B cells entering apoptosis, but is also exploited by the Human Immunodeficiency Virus (HIV), or Shiga Toxin from *Shigella dysenteriae* (Lindberg et al., 1987; Mangeney et al., 1991; Hammache et al., 1999). Interestingly, the two glycosphingolipids are differentially expressed depending on the cell-cycle with an upregulation of GM1 in G0/G1 phase and increased expression of Gb3 in G2/M phase (Majoul et al., 2002).

Here, we first set out to investigate the mobility of CD147 upon *N. meningitidis* infection by single-molecule tracking experiments. Next, we investigated the role of the two sphingolipids GM1 and Gb3 during infection with *N. meningitidis* using fluorescently labeled CTxB and STxB subunits, respectively. Super-resolution microscopy by structured illumination microscopy (SIM) (Gustafsson, 2000) and *direct* stochastic optical reconstruction microscopy (*d*STORM) (Heilemann et al., 2008; van de Linde et al., 2011) shows GM1 accumulation around meningococci highlighting their significant importance for bacterial invasion.

## Materials and Methods

### Bacterial Strains

*Neisseria meningitidis* strain MC58 was used in this study as a representative strain. Strain MC58 is a serogroup (Sg) B strain of the sequence type (ST)-74 (ST-32 clonal complex [cc]), which was isolated in 1983 in the United Kingdom and was kindly provided by E. R. Moxon (McGuinness et al., 1991). *N. meningitidis* strain 8013 (clone 12, also designated 2C43) was used for the preparation of the pili enriched fraction (PeF). This strain is a piliated capsulated Opa-, Opc- variant of the serogroup C meningococcal clinical isolate 8013 (ST-77/ST-8 clonal complex (cc), Institut Pasteur, 1989) and was kindly provided by M. Taha (Nassif et al., 1993). *N. meningitidis* strains were grown overnight on Columbia blood agar plates (bioMérieux) at 37°C and 5% CO_2_ in a humidity incubator and cultured on the next day in PPM + medium (proteose-peptone medium supplemented with 1× Kellogg’s supplement, 0.01 M MgCl_2_ and 0.005 M NaHCO_3_).

### Cell Culture

Immortalized human brain microvascular endothelial cells (HBMEC) were kindly provided by K. S. Kim (Stins et al., 1997) and were cultured as described previously (Unkmeir et al., 2002). Briefly, cells were cultured in RPMI-1640 medium supplemented with 1% sodium pyruvate (1 mM), 1% L-glutamine (2 mM), 1% non-essential amino acids (all purchased from GE Healthcare, Little Chalfont, United Kingdom), 5 U/ml heparin (Biochrom, Berlin, Germany) and 30 μg/ml endothelial cell growth supplement (ECGS, CellSystems, Troisdorf, Germany). Cells were incubated at 37°C and 5% CO_2_ in a humidified atmosphere.

### Infection Assays

Adhesion and invasion was determined by using the gentamicin protection assays as described elsewhere (Simonis et al., 2014). Briefly, cells between the 10th and 25th passages were used for infection assays at a density of 4 × 10^5^ cells/well. Cell medium was changed to infection medium [RPMI + 10% human serum (HS)] and cells were infected with MC58 at a multiplicity of infection (MOI) of 100 for 4 h. If indicated, cells were pre-incubated with 6.6 μg/ml CTxB in RPMI for 30 min prior to the medium change. To determine the number of adherent bacteria, cells were washed three times with phosphate buffered saline (PBS), to remove unbound bacteria, and afterward incubated with 1% saponin in RPMI to lyse the cells. Then, the cell-lysates were collected, diluted and plated on blood agar plates. To determine invasive bacteria, cells were handled similar to the adherent set with the exception of an additional incubation of the cells with gentamicin (200 μg/ml) for 2 h prior to the saponin treatment to kill all extracellular bacteria.

### Immunofluorescence and Fluorescence Microscopy

HBMEC were seeded onto 0.2% gelatine coated 8-well chamber slides (Sarstedt) at a density of 2 × 10^4^ cells/well and incubated for at least 24 h. To avoid possible interference of labeled CD147 receptors with the coating during single-molecule tracking, HBMEC were seeded onto KOH cleaned 8-well glass instead. Following infection with the indicated bacterial strain, cells were fixed with 2% formaldehyde and 0.2% glutaraldehyde for 15 min and washed. Labeling was performed with CTxB and/or STxB (Sigma-Aldrich, custom conjugated to Alexa Fluor 647 or Alexa Fluor 555) at a concentration of 5 μg/ml for 1 h. Alternatively, cells were incubated over night with Alexa Fluor 488 phalloidin as recommended by standard protocols (Thermo Fisher Scientific). To immobilize the toxin subunits, cells were again fixed by 2% formaldehyde and washed with PBS before *d*STORM imaging. Samples were embedded in prolong glass antifade mountant for SIM (Zeiss Elyra S.1) or covered with switching buffer (100 mM Cysteamine in PBS, pH 7.7) for *d*STORM. Imaging conditions and microscope setups were used as previously described (Burgert et al., 2017). Reconstruction from the raw data was performed with ThunderSTORM (Ovesný et al., 2014) or Zeiss ZEN software for *d*STORM and SIM, respectively. Spatial analysis of localization data was done with custom-made Python software. Ripley’s h function was computed and analyzed as described in Burgert et al. (2017). Ripley’s h function (Kiskowski et al., 2009) was computed for experimental and simulated data. Synthetic data with a localization density and region equal to the experimental data was prepared from a homogeneous point process of complete spatial randomness and from a clustered Neyman-Scott point process in which parent localizations are homogeneously distributed and accompanied by normal-distributed child localizations. The number of child localizations is Poisson distributed with a mean equal to the number of localizations per cluster as found for the experimental data sets. The standard deviation of the normal distribution is set to 8 nm resembling the localization precision. Ripley’s h function was computed 100 times from 200 random data points in order to estimate the variance.

### Single-Molecule Tracking

To perform single-molecule tracking, the non-competitive monoclonal CD147 antibody (clone: MEM-M6/1, Biorad) was directly coupled to the amine-reactive dye SeTau-647-NHS (SETA Biomedicals) to obtain a degree of labeling of 1.7. After purification by size-exclusion (Zeba spin desalting columns 40K MWCO Thermo Fisher Scientific) HBMEC were labeled with 0.17 nM antibody solution for 5 min at 37°C and washed twice before imaging in FluoroBrite DMEM media (Thermo Fisher Scientific). If stated, HBMEC were incubated for 30 min with 10 μg/ml MEM-M6/6 CD147 antibody or 2 μg PeF/well before labeling with MEM-M6/1 antibody. Imaging was performed at the setup described in the microscopy methods section with exposure time 20 ms for a total acquisition time of 100 s. Spot detection was performed by fitting with ThunderSTORM (Ovesný et al., 2014) and tracks generated and filtered for minimal track length of 20 frames with the Python implementation of the Crocker-Grier (Crocker and Grier, 1996) algorithm Trackpy (Allan et al., 2016). Mean squared displacements of each measurement were calculated and the resulting ensemble MSD was fit with a power law (Manzo and Garcia-Parajo, 2015; Shen et al., 2017), MSD(τ) = *αt^*n*^*, yielding the distribution of the generalized diffusion constant [α] and anomalous exponent [n].

### G1 Synchronization of HBMEC

G1 synchronization was performed using the method of serum starvation. 24 h prior to the experiment, HBMEC growth medium was removed and cells were washed once with PBS. Afterward, RPMI was added and the cells were further incubated as mentioned bevor. The cell population shift was controlled by propodium iodid (PI) staining. For that, cells were washed once with PBS and harvested in Eppendorf tubes. Afterward cells were washed three times with PBS, fixed in 3.7% formaldehyde for 30 min on ice and permeabilized with 0.25% Triton X-100 in PBS on ice. Cells were then stained with 10 μg/ml PI + 25 μg/ml RNase and incubated for 30 min at room temperature in the dark and immediately analyzed afterward. 10,000 cells were analyzed using the FACSCalibur^TM^ flow cytometer (BD Bioscience) and data were analyzed and graphed using FlowJo v10 (FlowJo, LLC). The gating strategy for G1, S, and G2 phase is shown in Supplementary Figure S5B.

### Flow Cytometry

Three days prior to the experiment, 1.25 × 10^5^ cells/ml were seeded in a 24-well plate and grown to approximately 1 × 10^6^ cells/ml. On the day of the infection experiment, cell medium was replaced by RPMI + 10% HS. Cells were infected with bacteria for 4 h. After infection, cells were washed once with PBS, trypsinized and harvested in an Eppendorf tube. After washing with ice cold FACS buffer (5% FCS in PBS), cells were incubated with Alexa Fluor 647 labeled CTxB for 30 min at room temperature in the dark. After incubation, cells were washed three times with FACS buffer and fixed in 3.7% paraformaldehyde in PBS for 30 min at 4°C. Afterward, cells were washed three times with FACS buffer and 500 μl were transferred into a FACS-tube for the measurement. 10,000 cells were analyzed using the FACSCalibur^TM^ flow cytometer (BD Bioscience) and data were analyzed and graphed using FlowJo v10 (FlowJo, LLC).

### Preparation of Pilus Enriched Fractions (PeF)

Pilus enriched fractions (PeF) were prepared as described previously (Peters et al., 2019). The bacterial content of 50 blood agar plates was harvested in 40 ml of 0.15 M ethanolamine (in PBS) with a pH of 10.5. Pili were sheared of by intensive vortexing for 2 min followed by centrifugation at 12.000 × *g* for 10 min at room temperature to remove cellular debris. The supernatant was used for an additional centrifugation step at 21.000 × *g* for 90 min to remove smaller debris. Then, the supernatant was transferred to an Erlenmeyer flask and ammonium sulfate saturated 0.15 M ethanolamine was added to a concentration of 10% and was incubated under continuous shacking for 30 min at room temperature. The protein-ammonium sulfate precipitate was then harvested by centrifugation at 21.000 × *g* for 15 min. The supernatant was discarded and the pellet was re-suspended in 0.05 M Tris buffer saline (TBS) pH 7.5. Protein solutions were then applied to a 6 ml Viva Spin column with a 7 kDa molecular weight cut of (MWCO) and were centrifuged at 4000 × *g* at room temperature until the volume reaches 1 ml. To clean the sample, TBS was added again to 6 ml followed by centrifugation as mentioned above.

### Statistical Analysis and Data Visualization

Statistical analysis was performed by either unpaired two-tailed Student’s *t*-test or analysis of variance (ANOVA) test followed by a *post hoc* test. Significance values are indicated by asterisks: ^∗^*P* < 0.05; ^∗∗^*P* < 0.01; ^∗∗∗^*P* < 0.001; ^****^P < 0.0001. Normality was tested using the Kolmogorov–Smirnov test. Data was visualized as box plots showing the interquartile range (IQR) of the data with median as line and mean as square. The whiskers represent the lowest and highest value within 1.5 IQR of the lower and upper quartile, respectively. Outliers are shown as filled squares outside the IQR box.

## Results

### Single-Molecule Tracking Reveals Modulation of CD147 Receptor Mobility Upon Interaction

**FIGURE 1:**
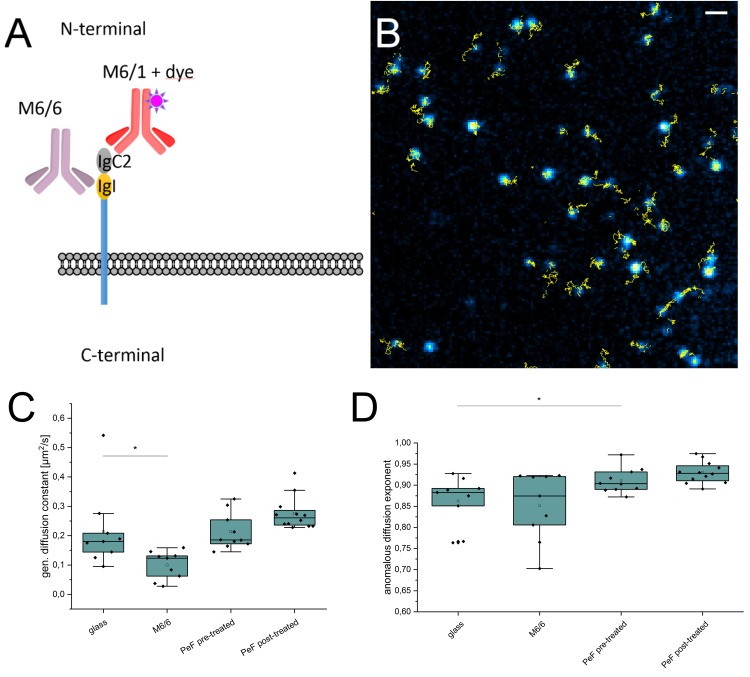
Single-molecule tracking of CD147 on HBMEC using monoclonal antibodies. **(A)** CD147 is a single pass membrane protein with two extracellular Ig-like domains. The N-terminal IgC2 domain is recognized by the MEM-M6/1 antibody which was conjugated to the photostable dye SeTau-647 (Tsunoyama et al., 2018) and used for single-molecule tracking. The membrane proximal IgI domain is the binding site for MEM-M6/6 and type IV pili of *N. meningitidis*. **(B)** Example of CD147 single-molecule tracks. SeTau-647 coupled MEM-M6/1 is depicted in cyan with corresponding local tracks in yellow. Overlay was created with the Fiji (Schindelin et al., 2012) plugin TrackMate (Tinevez et al., 2017). Scale bar, 1 μm. **(C)** The diffusion coefficient of CD147 is reduced upon pretreatment with 10 μg/ml MEM-M6/6 and hardly affected by PeF treatment. Values represent individual single-molecule tracking experiments. **(D)** The anomalous diffusion exponent is not affected by MEM-M6/6 treatment but increases slightly upon PeF treatment. Significance values are indicated by asterisks: ^∗^*P* < 0.05; ^∗∗^*P* < 0.01; ^∗∗∗∗^*P* < 0.001; ^∗∗∗∗∗∗^*P* < 0.0001.

It has been shown that CD147 and β2-adrenergic receptor (ß2AR) are organized in pre-existing complexes at the plasma membrane of endothelial cells, which accumulate at sites of meningococcal adhesion (Maïssa et al., 2017). This local enrichment of CD147-ß2AR complexes in the plasma membrane possibly allows bacteria to adhere to vascular walls *in vivo* and resist hemodynamic forces of blood flow. Since accumulation of receptors at bacterial adhesion sites requires a high mobility in the plasma membrane we performed live-cell single-molecule tracking experiments of CD147 under various experimental conditions using an N-terminal binding monoclonal antibody (MEM-6/1) directly conjugated to the photostable fluorescent dye SeTau-647 (Tsunoyama et al., 2018). In contrast to the membrane-proximal binding monoclonal antibody MEM-6/6 (Figure 1A) MEM-6/1 does not compete with the binding site of type IV pili as demonstrated by single-molecule tracking experiments of human brain microvascular endothelial cells (HBMEC) pretreated with saturating concentrations of pilus enriched fraction (PeF) (Figure 1A and Supplementary Figure S1; Bernard et al., 2014). Pretreatment with saturating PeF concentration did significantly reduce the number of accessible MEM-M6/1 epitopes during individual single-molecule tracking experiments (Supplementary Figure S1).

Using SeTau-647 labeled MEM-6/1 antibodies we followed CD147 on the basal plasma membrane of human brain microvascular endothelial cells (HBMEC) for a duration of 100 s with a time resolution of 20 ms using total internal reflection fluorescence (TIRF) microscopy (Figure 1B and Supplementary Video S1). For quantification of diffusion dynamics, we analyzed the mean square displacement (MSD) and fitted it with a power law (Manzo and Garcia-Parajo, 2015; Shen et al., 2017):

$$\text{MSD}\,\left(\tau \right)=\alpha \,\tau^{n}$$

Treatment of HBMEC with the competitive MEM 6/6 antibody reduced the generalized diffusion constant α (Figure 1C) as well as the number of localized CD147 molecules (Supplementary Figure S1) whereas the anomalous diffusion exponent *n* remained unaltered (Figure 1D). Addition of meningococcal PeF before (pre-treated) and after labeling (post-treated) increased α and *n* only slightly (Figures 1C,D) demonstrating that PeF does not significantly change the mobility of the neisserial type IV pili receptor CD147. Still, the slight changes in mobility observed may indicate cytoskeletal rearrangements of the plasma membrane sphingolipid organization. Indeed a recent study revealed an increase in ceramide-rich platforms upon treatment of HBMEC with type IV pili (Peters et al., 2019). Therefore, we investigated the distribution and localization of the native glycosphingolipids GM1 and Gb3 by super-resolution microscopy.

### Rearrangement of Plasma Membrane Sphingolipids During Meningococcal Infection

**FIGURE 2:**
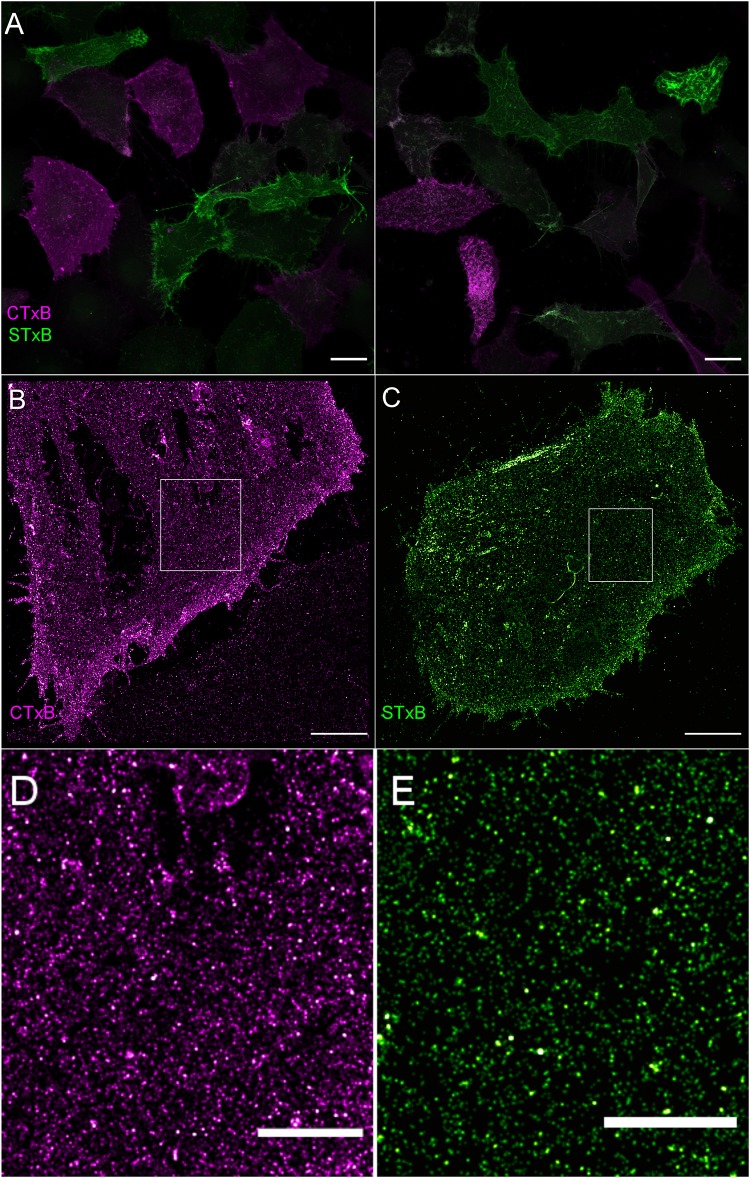
Visualization of sphingolipids GM1 and Gb3 in the plasma membrane of brain endothelial cells. **(A)** Confocal laser scanning microscopy images of GM1 (magenta) and Gb3 (green) labeled with CTxB-Alexa Fluor 647 (magenta) and STxB-Alexa Fluor 555 (green), respectively. Scale bar, 20 μm. **(B)** 2D dSTORM images of GM1 labeled with CTxB-Alexa Fluor 647, and **(C)** Gb3 labeled with STxB-Alexa Fluor 647 showing a homogeneous distribution of the two sphingolipids in the plasma membrane of HBMEC. Scale bar, 5 μm. **(D)** Expanded views of the white boxed regions showing homogeneous distributions of CTxB **(D)** and STxB **(E)**. Both regions are representative areas which were used for cluster analysis by Ripley’s h function (Supplementary Figure S2). Scale bar 2 μm.

To investigate possible changes in lipid organization upon meningococcal adhesion we visualized the distribution of the two sphingolipids GM1 and Gb3 in the plasma membrane of brain endothelial cells using the cholera toxin B (CTxB) and shiga toxin B (STxB) subunit, respectively. Two-color confocal laser scanning images of HBMEC show that GM1 and Gb3 exhibit cell-cycle dependent expression rates (Figure 2A), only in S phase both sphingolipids are expressed and simultaneously detectable in the plasma membrane (Majoul et al., 2002). Corresponding *d*STORM images show that GM1 and Gb3 are homogeneously distributed throughout the plasma membrane of HBMEC (Figures 2B–D) without clear indication of clustering (Supplementary Figure S2). Analysis of the spatial distribution of localization data by Ripley’s h function indicates merely clusters on length scales similar to the *d*STORM localization precision (∼8 nm). These clusters originate from repeated detection of fluorophores on each toxin subunit. The number of localizations per cluster (as quantified by the DBSCAN clustering algorithm) follows the degree of labeling of pentameric CTxB (0.9) and STxB (0.5). The same observations were made on toxin subunits unspecifically bound to the glass surface (Supplementary Figure S3).

**FIGURE 3:**
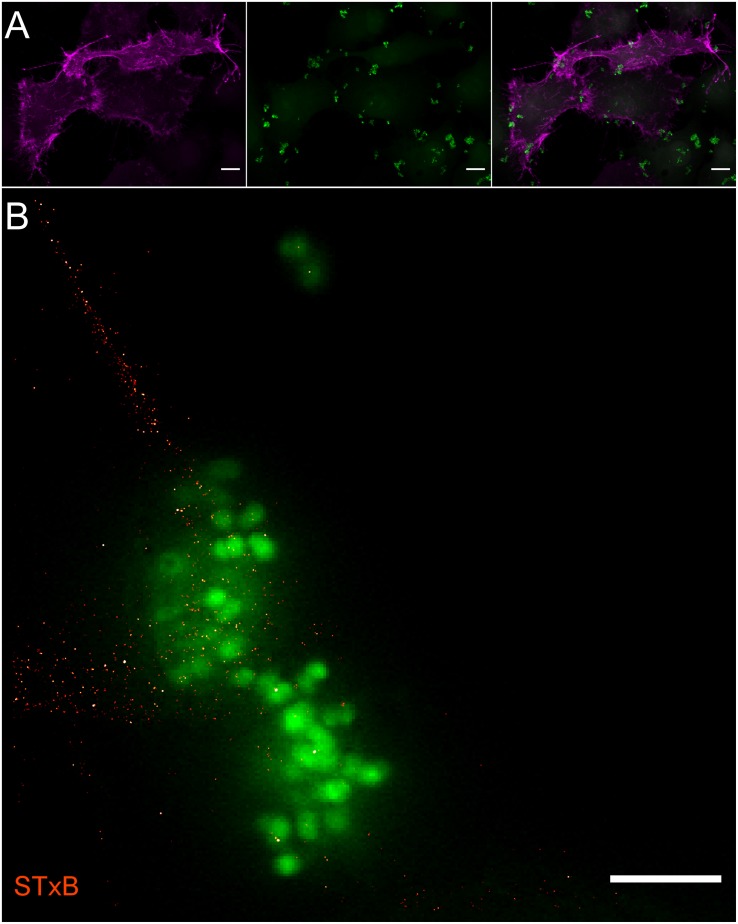
Fluorescence microscopy of Gb3 in the plasma membrane of brain endothelial cells during *N. meningitidis* infection. **(A)** Confocal laser scanning microscopy of HBMEC labeled with STxB-Alexa Fluor 555 (magenta) and infected with GFP expressing *N. meningitidis* (green). No accumulation of Gb3 was observed at invasion sites. Scale bar, 5 μm. **(B)** Super-resolution *d*STORM image showing that the distribution of Gb3 in the plasma membrane of HBMEC does not change upon infection with meningococci (green). Gb3 was labeled with Alexa Fluor 647 conjugated STxB (red). The diffraction limited GFP signal was upscaled for the overlay. Same imaging and analysis parameters were used for the *d*STORM image as in Figure 2. Scale bar, 5 μm.

**FIGURE 4:**
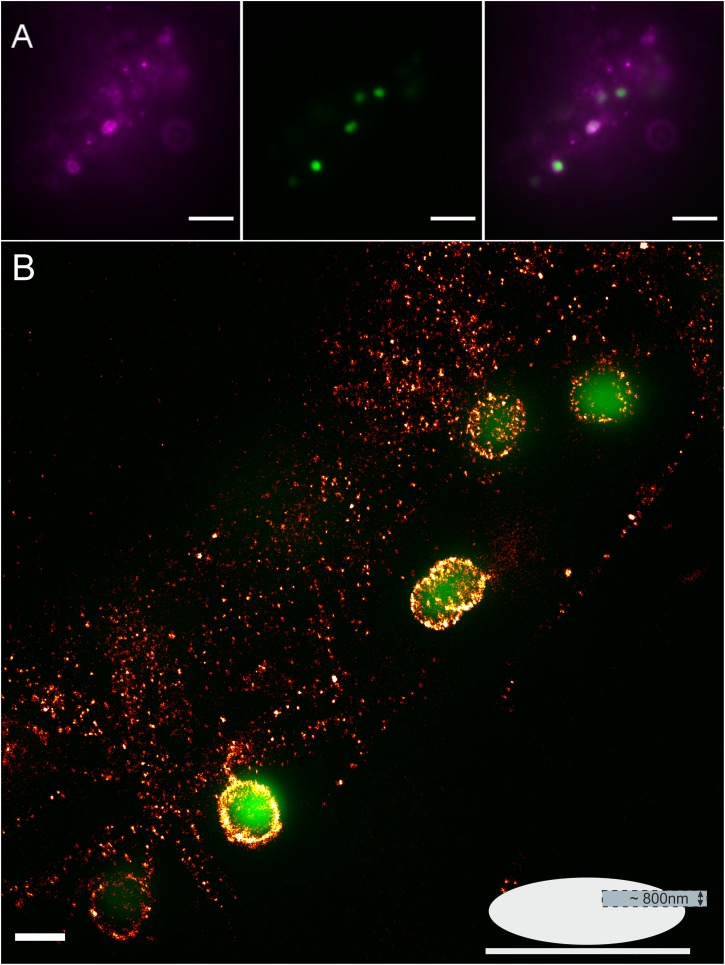
Fluorescence imaging of GM1 in HBMEC upon infection with GFP expressing *N. meningitidis*. **(A)** Widefield fluorescence images of meningococci (green) and GM1 labeled with Alexa Fluor 647 CTxB (magenta). Scale bar, 5 μm. **(B)** *d*STORM image showing strong accumulation of GM1 around infection sites. The lower right corner shows a schematic model illustrating the mode of imaging. Adherent HBMEC were irradiated by epi-illumination. Detection was performed in an axial plane ensuring the imaging of a substantial part of the plasma membrane (light blue area). The axial area captured by the 2D *d*STORM image is determined to approximately 800 nm by the blurring of the point spread function above and below the imaging plane and data analysis parameter. Scale bar 1 μm.

Upon infection of cells with *N. meningitidis* the plasma membrane distribution of Gb3 remained unchanged (Figures 3A,B). In contrast GM1 showed a remarkable increase in fluorescence intensity around adhesive meningococci on the plasma membrane of HBMEC (Figure 4A). *d*STORM images of CTxB labeled HBMEC in the presence of meningococci were recorded from an axial plane slightly above the equatorial plane under epi-illumination to ensure imaging of a large part of the cellular plasma membrane with adhesive bacteria (Figure 4B). Our data clearly demonstrate strong accumulation of the ganglioside GM1 around adhesive bacteria (Figure 4B) whereas uninfected HBMEC show a homogeneous distribution of GM1 in the plasma membrane (Figure 2). To exclude non-specific binding of CTxB and STxB to meningococci, bacteria were seeded onto glass without HBMEC, labeled and imaged by *d*STORM. The corresponding images show that the two sphingolipids do not bind non-specifically to meningococci (Supplementary Figure S3).

Next, we tested if CD147 and actin as highly conserved key cytoskeletal protein involved in organization of the plasma membrane, colocalize with GM1 and accumulate around meningococci adhesion sites on the plasma membrane of HBMEC (Coureuil et al., 2010; Maïssa et al., 2017). However, SIM images show strong colocalization of the adhesion receptor CD147 and actin but no enrichment or morphological change at invasion sites of bacteria (Supplementary Figure S4).

### Increased Bacterial Invasion Upon G1 Phase Arrest and GM1 Upregulation

Since the expression of GM1 and Gb3 is highest in G1 and G2 phase of the cell cycle, respectively (Majoul et al., 2002) we next investigated cell cycle dependent effects on the adhesion and invasion efficiency of *N. meningitidis*. Serum starvation 24 h before the experiment caused a significant increase of HBMEC residing in G1 phase as demonstrated by PI staining of the DNA and flow cytometry analysis (Supplementary Figures S5A,B). Simultaneously the concentration of ganglioside GM1 present in the plasma membrane in G1 phase increased substantially (Supplementary Figure S5C).

**FIGURE 5:**
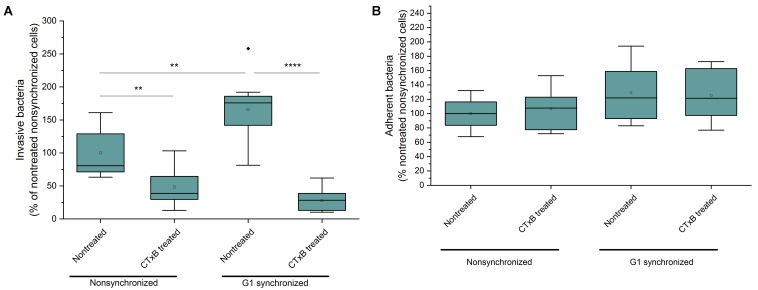
Adhesion and invasion efficacy of *N. meningitidis* upon cell cycle arrest of HBMEC in G1 phase and CTxB pre-treatment. **(A)** The number of invasive meningococci was determined by gentamicin protection assays. Here, all extracellular bacteria were killed by incubation for 2 h in gentamicin solution and intracellular bacteria counted. Non-synchronized or G1 synchronized cells were preincubated with 6.6 μg/ml CtxB for 30 min at 37°C (if indicated) and afterward infected with MC58 for 4 h with a MOI of 100. **(B)** The number of adhesive bacteria was determined by counting of residual meningococci colonies after thorough washing and lysis of HBMEC.

Interestingly, infection of G1 phase arrested cells caused an even more pronounced increase of GM1 levels present in the plasma membrane of HBMEC (Supplementary Figure S5C). To analyze effects of increased GM1 levels during G1 phase on bacterial adhesion and invasion we performed gentamicin protection assays to estimate the number of adherent or invasive bacteria by counting of residual bacterial colonies. Here, bacterial adhesion was neither influenced by serum starvation of host cells nor blocking of GM1 by unlabeled CTxB (Figure 5B). In contrast, invasion of HBMEC by meningococci was significantly increased in synchronized cells and this effect could be abolished by blocking of GM1 (Figure 5A).

## Discussion

Single-molecule tracking enables the observation of highly dynamic processes from viral cell entry mechanisms (Ruthardt et al., 2011) to ligand-binding (Yanagawa et al., 2018) at high spatiotemporal resolution. Upon ligand-binding and subsequent activation, receptors typically undergo conformational changes and/or changes in oligomerization states, which is often accompanied by reduced mobility resulting in decreased diffusion coefficients (Chung et al., 2010; Yanagawa et al., 2018). In this study, we could show that the presence of a PeF alone did not significantly change the diffusion coefficient of neisserial type IV pilus receptor CD147. Rather, our data indicate a slightly altered type of mobility toward normal diffusion which might indicate cytoskeletal rearrangements or modulation of the plasma membrane lipid environment. Interestingly, addition of the monoclonal M6/6 antibody before single-molecule tracking experiments resulted in a strong decrease in the number of M6/1 antibodies bound on the plasma membrane.

Since both antibodies are capable to bind to monomeric and dimeric CD147 molecules (Koch et al., 1999) M6/6 antibody-induced clustering resulting in a reduced M6/1 antibody epitope accessibility can be excluded as explanation. Instead, the following hypotheses seem to be more plausible. Binding of M6/6 to the membrane proximal Ig-like domain might activate the receptor and induce the local production of matrix metalloproteinases leading to subsequent receptor shedding. Indeed, CD147-induced expression of matrix metalloproteinases results in proteolytic cleavage of membrane-associated CD147 and an increase of its soluble form (Tang et al., 2004). Additionally, the diffusion coefficient of CD147 was significantly reduced upon M6/6 antibody binding indicating that activation of CD147 reduces its mobility in the plasma membrane (Figure 1C). Notably, the M6/6 antibody has unique properties and can inhibit OKT3-induced T cell proliferation (Koch et al., 1999) or modulate multidrug resistance (Somno et al., 2016). This implies that CD147 signaling might influence plasma membrane organization and promote immobilization of the receptor. For this reason, following studies should dissect effects introduced by specific binding characteristics of the antibodies by using monovalent Fab fragments.

In contrast, addition of PeF did slightly increase the mobility of CD147 receptors (Figure 1D) although neisserial type IV pili and the M6/6 antibody compete for the same binding site (Bernard et al., 2014). In general, the affinity of the PilE and PilV monomers to CD147 is low and the need for multimeric organization as type IV pili seems to play an important role in mediating adherence (Bernard et al., 2014). Since our pili preparation contains mainly monomeric pilin subunits, as shown by Peters et al. (2019), incubation with our PeF preparation might not resemble the native condition where in addition to the multimeric assembly as pilus fibers whole micrometer-sized bacteria are attached to CD147. It seems thus more likely, that binding of the competitive M6/6 antibody reflects the native interaction of type IV pili with CD147 although this has to be verified in future experiments.

Glycosphingolipids represent important pathogen receptors (Nakayama et al., 2018) with thousands of possible structures. Notably, bacterial lipopolysaccharides are able to mimic host cell glycosphingolipids causing evasion of the immune system or leading to autoimmune diseases (Harvey et al., 2001). Although several possible host glycosphingolipids binding partners have been identified for *N. meningitidis* (Hugosson et al., 1998; Mubaiwa et al., 2017) molecular information about their involvement in pathogen interactions remained elusive. Furthermore, with a bacteria size of approximately 1 μm, the molecular details of host-bacteria interactions are difficult to image with conventional diffraction-limited fluorescence microscopy. Using single-molecule sensitive *d*STORM we could show that gangliosides are important host cell receptors mediating cellular entry of meningococcus by accumulating at bacterial adhesion sites (Figure 4B). Here, it has to be considered that CTxB does not exclusively bind to GM1 but possibly also to a plethora of other gangliosides (Kuziemko et al., 1996). Upon binding CTxB can be endocytosed via caveolae and clathrin-independent pathways although clathrin-mediated endocytosis seems to cover the major fraction (Torgersen et al., 2001). Which pathways are used in the context of meningococcal invasion and whether the bacteria are able to locally induce upregulation of GM1 or if this is a passive event triggered by cell cycle modulation is presently unknown and requires further experiments. Of note, pentameric STxB and CTxB possess multiple binding sites for individual glycosphingolipids and binding can be influenced by the chain length and saturation state of the attached fatty acid (Pellizzari et al., 1992; Kiarash et al., 1994). In order to reduce possible effects induced by multivalent toxin binding we fixed the cells before labeling to immobilize the binding partners.

**FIGURE 6:**
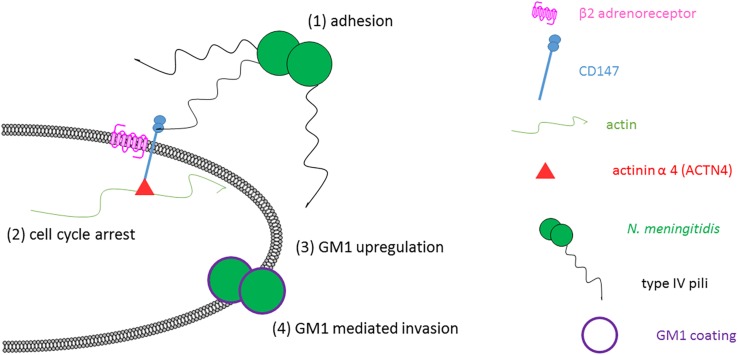
Hypothetic model of meningococcal host cell invasion. *N. meningitidis* adhere with type IV pili to the membrane proximal Ig-like domain of CD147. After enrichment of β2-adrenergic receptor and actin downstream signaling cascades are triggered causing cell cycle arrest in G1 to S phase. Cell cycle dependent upregulation of GM1 at the plasma membrane increases the invasion efficacy of *N. meningitidis*. Bacteria interact with plasma membrane GM1 gangliosides facilitating entry into the cell or evasion from the human immune system. Drawing not to scale.

However, our findings demonstrate that cell cycle arrest in G1 phase causes an increase of plasma membrane GM1 molecules leading to enhanced bacterial uptake. Blocking of GM1 strongly reduces infection efficiency implying the importance of plasma membrane gangliosides for bacterial invasion. *N. meningitidis* infection can cause accumulation of brain endothelial cells in S phase (Oosthuysen et al., 2016) and of human epithelial cells in G1 phase (Papen et al., 2016) and both cell cycle phases are positive for CTxB labeling (Majoul et al., 2002). Therefore, we propose a model where meningococci regulate their own uptake by initiating a positive feedback loop (Figure 6). The increased invasion efficacy should thus even be more pronounced in human epithelial cells whose gangliosides have already been described to interact with *Pseudomonas aeruginosa* pili (Comolli et al., 1999). We assume that this mechanism might play an important role in the initial uptake from the nasopharynx into the blood. Blocking this interaction could represent a promising method to avoid life-threatening dissemination of meningococci and help to develop therapeutic approaches for bacterial clearance.

## Data Availability

The raw data supporting the conclusions of this manuscript will be made available by the authors, without undue reservation, to any qualified researcher.

## Author Contributions

JS designed and performed the experiments, applied the data analysis, and wrote the manuscript. SP performed the experiments involving living *N. meningitidis*, analyzed the data, and wrote the manuscript. SD performed the cluster analysis and data simulation, and provided the discussion. AS-U and MS guided the project, developed concepts, and wrote the manuscript.

## Conflict of Interest Statement

The authors declare that the research was conducted in the absence of any commercial or financial relationships that could be construed as a potential conflict of interest. The handling Editor declared a past co-authorship with one of the authors MS.

## Supplementary Material

FIGURE S1 Number of localizations detected during individual CD147 single-molecule tracking experiments on HBMEC. CD147 was labeled with subnanomolar concentrations of monoclonal CD147 MEM-M6/1 antibody conjugated to SeTau647. Localizations were detected by fitting with ThunderSTORM (Ovesný et al., 2014). HBMEC were seeded on KOH cleaned glass and pre-treated with 10 μg/ml MEM-M6/6 CD147 antibody or 2 μg PeF/well before labeling with MEM-M6/1 antibody, if stated.

FIGURE S2 Spatial distribution analysis of dSTORM data (red line). Ripley’s h function was estimated 100 times (gray data curves with mean shown in color) for the dSTORM data representing GM1 labeled with CTxB-Alexa647 (left) and Gb3 labeled with STxB-Alexa647 (right) as shown in Figures 2C,D, respectively. For comparisn, Ripley’s h function is shown for simulated data that represents a point process of complete spatial randomness (blue) and a clustered point process representing spatial clusters due to repeated localizations per toxin. The data indicates that experimental data is largely controlled by the photophysical clustering showing a homogeneous distribution of the two sphingolipids in the plasma membrane of HBMEC. The peak positions are found at nearly identical positions of (left) 27 nm and 22 nm and of (right) 38 nm and 21 nm for experimental and simulated data, representatively.

FIGURE S3 *d*STORM images of GM1 and Gb3 of GFP expressing *N. meningitidis* (green) without HBMEC labeled with Alexa Fluor 647 conjugated CTxB or STxB. Both toxins non-specifically bind to the coverslip but do not show any accumulation at bacteria. The diffraction limited GFP signal was upscaled for the overlay. Scale bar, 5 μm.

FIGURE S4 SIM images of GFP expressing meningococci infected HBMEC. CD147 labeled with monoclonal MEM-M6/1 and secondary F(ab)2-Alexa Fluor 647 (magenta). Actin labeled with phalloidin Atto565 (gray). GFP signal of meningococci (green) and overlay of the three images showing that CD147 and actin colocalize but do not accumulate at bacterial adhesion sites. Scale bar, 10 μm.

FIGURE S5 Flow cytometry analysis of cell cycle state and average ganglioside GM1 concentration present in the plasma membrane of HMBEC. **(A)** Serum starvation was performed 24 h prior to the experiment by replacing the growth medium with RPMI. On the next day, cells were harvested, fixed, permeabilized and stained with PI (10 μg/ml PI + 25 μg/ml RNase) for 30 min in the dark at room temperature. Afterward, PI incooperation was estimated via flow cytometry. Histogram of unsynchronized and G1 synchronized cells with the indicated gating strategy shown in **(B)**. **(C)** For the analysis of cell surface GM1, cells were stained with Alexa Fluor 647 conjugated CtxB for 30 min at room temperature in the dark. Then, cells were fixed for 30 min at 4°C, washed three times with FACS buffer and analyzed by flow cytometry.

VIDEO S1 Examples of CD147 single-molecule tracks measured from one cell.
