# Supplementary figures and images for: Super-Resolution Microscopy Reveals Local Accumulation of Plasma Membrane Gangliosides at Neisseria meningitidis Invasion Sites
Source: Front Cell Dev Biol. 2019 Sep 13;7:194. doi: 10.3389/fcell.2019.00194 (PMC6753371; doi:10.3389/fcell.2019.00194)

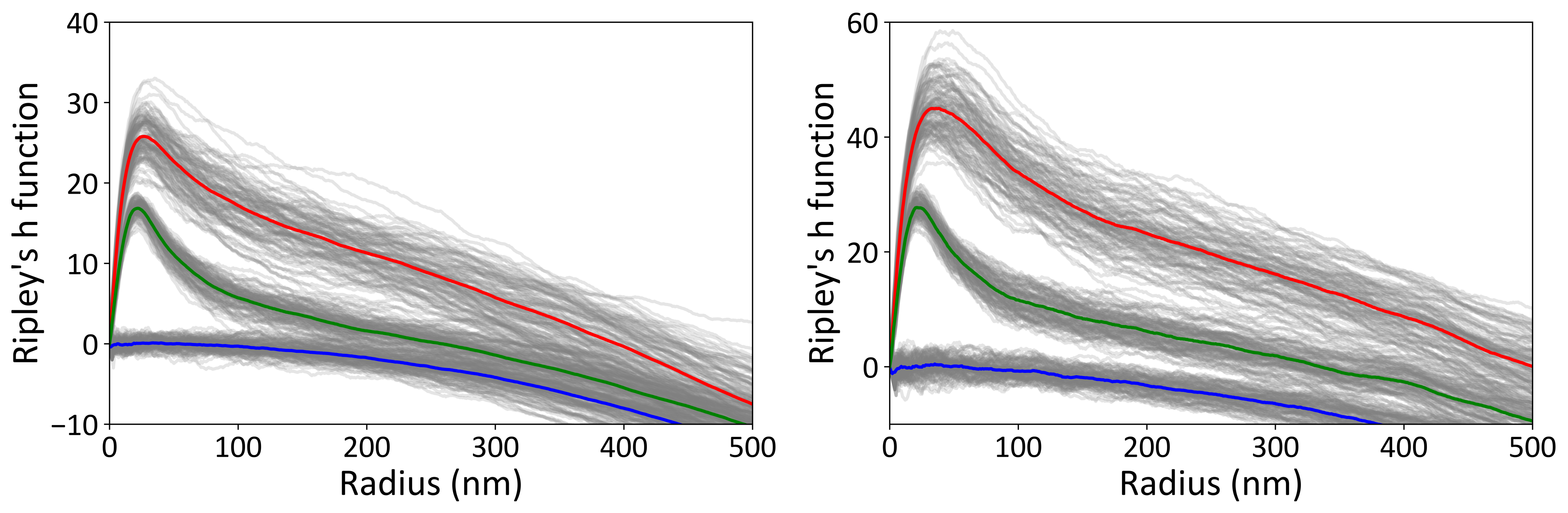

Supplement: FIGURE S2 — Spatial distribution analysis of dSTORM data (red line). Ripley’s h function was estimated 100 times (gray data curves with mean shown in color) for the dSTORM data representing GM1 labeled with CTxB-Alexa647 (left) and Gb3 labeled with STxB-Alexa647 (right) as shown in Figures 2C,D, respectively. For comparisn, Ripley’s h function is shown for simulated data that represents a point process of complete spatial randomness (blue) and a clustered point process representing spatial clusters due to repeated localizations per toxin. The data indicates that experimental data is largely controlled by the photophysical clustering showing a homogeneous distribution of the two sphingolipids in the plasma membrane of HBMEC. The peak positions are found at nearly identical positions of (left) 27 nm and 22 nm and of (right) 38 nm and 21 nm for experimental and simulated data, representatively. [file Image_2.TIF]

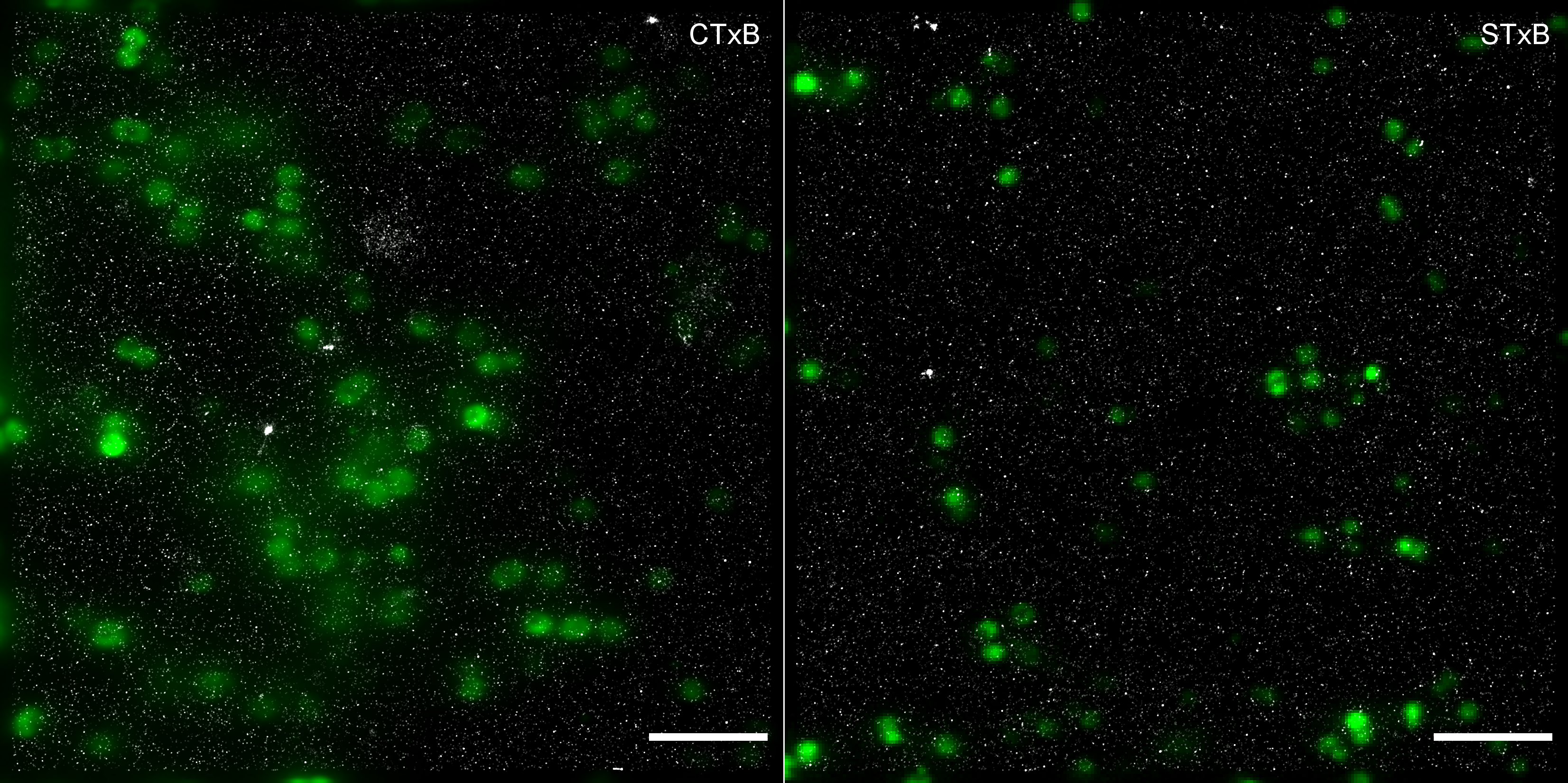

Supplement: FIGURE S3 — dSTORM images of GM1 and Gb3 of GFP expressing N. meningitidis (green) without HBMEC labeled with Alexa Fluor 647 conjugated CTxB or STxB. Both toxins non-specifically bind to the coverslip but do not show any accumulation at bacteria. The diffraction limited GFP signal was upscaled for the overlay. Scale bar, 5 μm. [file Image_3.TIF]

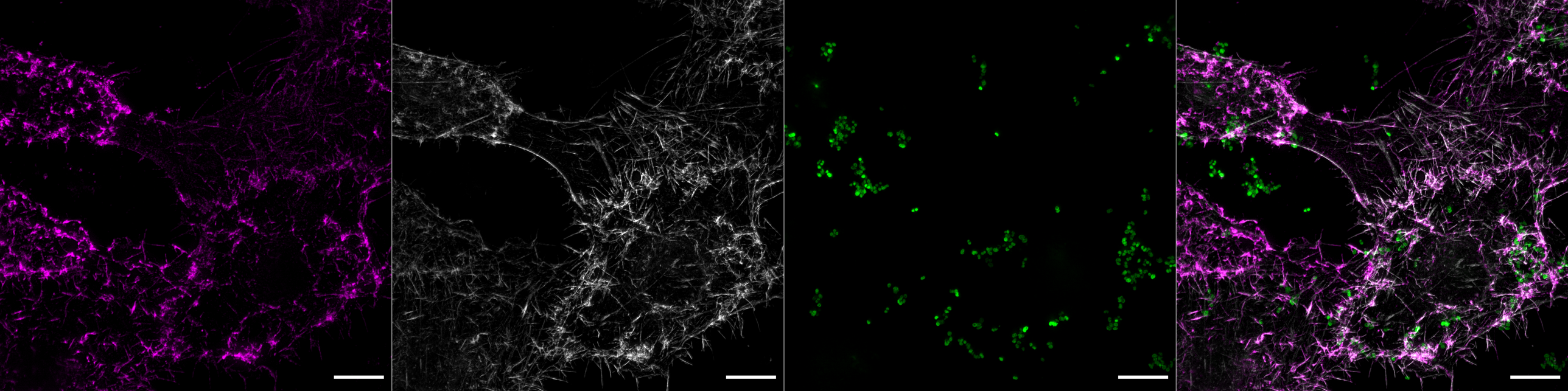

Supplement: FIGURE S4 — SIM images of GFP expressing meningococci infected HBMEC. CD147 labeled with monoclonal MEM-M6/1 and secondary F(ab)2-Alexa Fluor 647 (magenta). Actin labeled with phalloidin Atto565 (gray). GFP signal of meningococci (green) and overlay of the three images showing that CD147 and actin colocalize but do not accumulate at bacterial adhesion sites. Scale bar, 10 μm. [file Image_4.TIF]

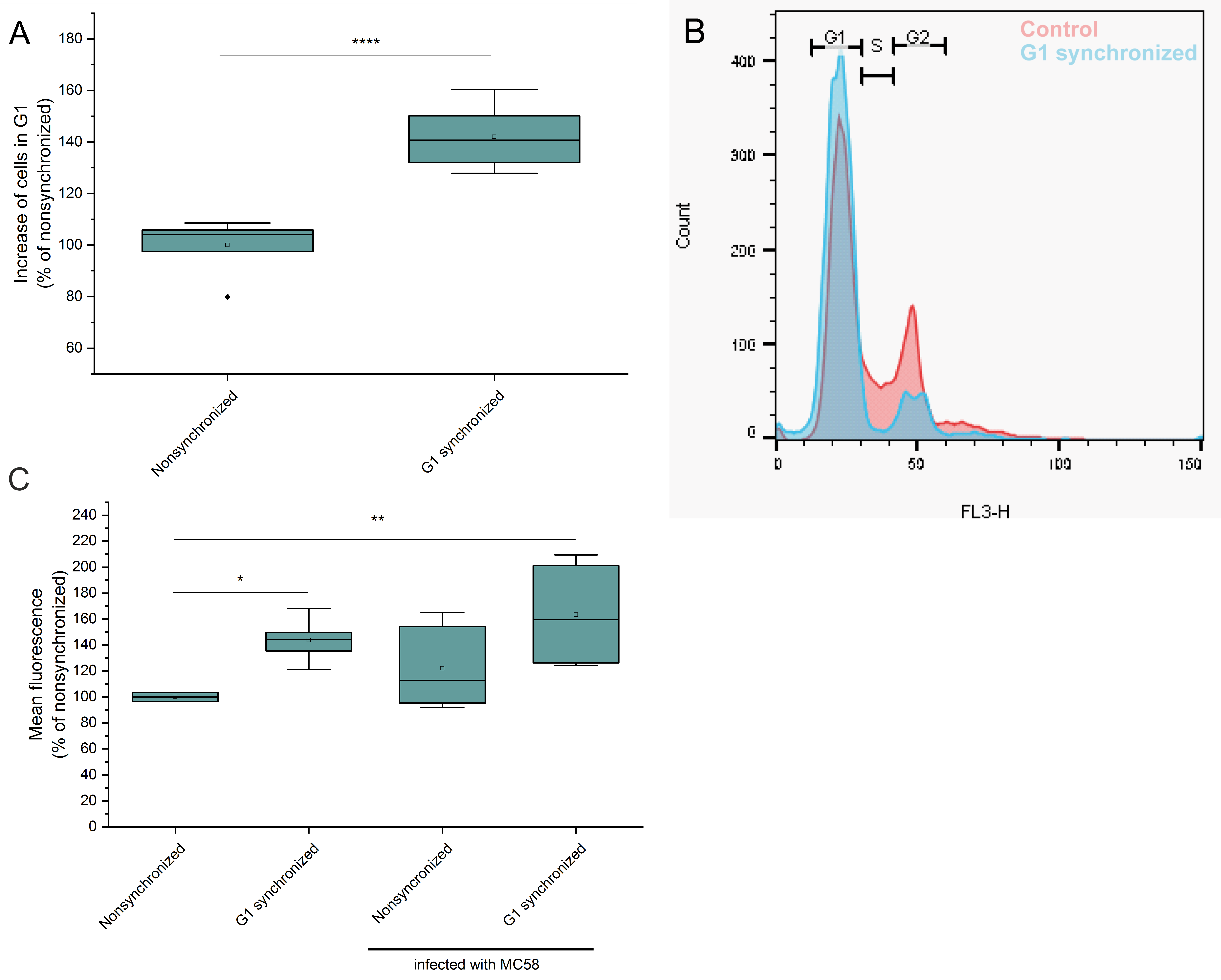

Supplement: FIGURE S5 — Flow cytometry analysis of cell cycle state and average ganglioside GM1 concentration present in the plasma membrane of HMBEC. (A) Serum starvation was performed 24 h prior to the experiment by replacing the growth medium with RPMI. On the next day, cells were harvested, fixed, permeabilized and stained with PI (10 μg/ml PI + 25 μg/ml RNase) for 30 min in the dark at room temperature. Afterward, PI incooperation was estimated via flow cytometry. Histogram of unsynchronized and G1 synchronized cells with the indicated gating strategy shown in (B). (C) For the analysis of cell surface GM1, cells were stained with Alexa Fluor 647 conjugated CtxB for 30 min at room temperature in the dark. Then, cells were fixed for 30 min at 4°C, washed three times with FACS buffer and analyzed by flow cytometry. [file Image_5.TIF]
